# First Report of Parkinsonism Associated With Indoximod, an Immune-Modulating Agent

**DOI:** 10.1200/JGO.2016.007492

**Published:** 2016-11-30

**Authors:** Mark Floyd, Badi El Osta, Shou-Ching Tang

**Affiliations:** **Mark Floyd**, **Badi El Osta**, and **Shou-Ching Tang**, Medical College of Georgia, Augusta University, Augusta, GA; and **Shou-Ching Tang**, Tianjin Medical University Cancer Institute and Hospital, Tianjin, People’s Republic of China.

## INTRODUCTION

A unique aspect of the toxicity profiles of immune checkpoint inhibitors (ICIs) is their tendency to trigger immune-related toxicities.^1^ Indoximod, an indoleamine 2,3-dioxygenase inhibitor, is a novel agent that upregulates effector T cells, which allows the host immune system to recognize and destroy tumor cells.^2,3^ Contrary to ICI, autoimmune toxicities have not yet been reported with indoximod. Here, we report the first case of indoximod-induced Parkinsonism in a patient with metastatic breast cancer who was enrolled in a clinical trial.

## CASE PRESENTATION, MANAGEMENT, AND OUTCOME

**Fig 1:**
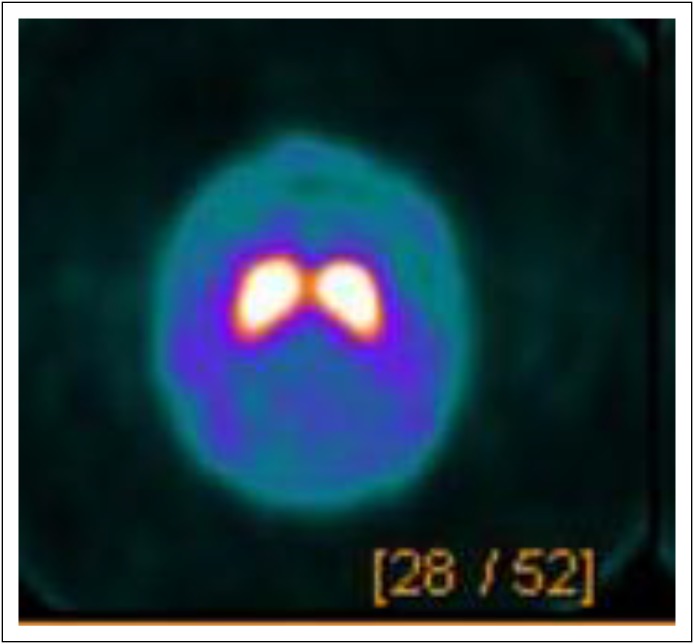
Brain SPECT DaTscan. Normal dopamine transporter uptake in the bilateral striata that favors drug-induced Parkinsonism.

A 61-year-old woman with metastatic breast cancer was enrolled in a phase II trial (NLG2101) in September 2015 and was randomly assigned to the experimental arm: docetaxel 75 mg/m^2^ administered intravenously every 3 weeks on day 8 plus indoximod 1200 mg oral on days 1 to 14. She tolerated cycles 1 and 2 and achieved a good response. In November, on day 16 of cycle 3, the patient developed severe fatigue and lower extremity weakness, without new back pain, and required a wheelchair for mobility. Home medications included aspirin, ibuprofen, oxycodone, pravastatin, ondansetron, prochlorperazine, ranitidine, alprazolam, calcium carbonate, vitamin B12 and D3, fish oil, and indoximod. Family history included Parkinson’s disease (PD) in her father. On exam, she was able to stand and walk with assistance only, had 4/5 strength in all extremities, a shuffling gait, no arm swing, resting tremor in her hands, rigidity, and a fixed facial expression. Bloodwork was normal and ruled out thyroid disease, adrenal insufficiency, or electrolyte abnormalities. Brain magnetic resonance imaging showed no evidence of progressive multifocal leukoencephalopathy or encephalitis. Within a week, she began having dysphagia and dysarthria and was evaluated by neurology. CSF and electromyogram were unrevealing and ruled out viral encephalitides and myositis or other myopathies, respectively. One week later, she developed hypophonia, slow ocular upward tracking and nonexistent downward tracking, upper extremity hypertonicity, and cogwheel rigidity. She was diagnosed with Parkinsonism, having the cardinal signs of resting tremor, rigidity, and bradykinesia, along with common signs of masked facies and shuffling gait, with other possible diagnoses ruled out. A SPECT DaTscan showed normal dopamine transporter uptake in bilateral striata (Fig 1), which was consistent with drug-induced Parkinsonism. None of the multiple medications she was taking has been found to cause Parkinsonism, and as indoximod is the only drug she was taking that had not been extensively studied, it is the likely culprit. Indoximod was discontinued and she was started on carbidopa-levodopa and trihexyphenidyl without any clinical improvement. She then received 6 weeks of high-dose prednisone with near resolution of her symptoms. Unfortunately, the patient subsequently died in May 2016 from cardiac arrest.

## DISCUSSION

To our knowledge, this is the first report of indoximod-induced Parkinsonism. We reported the preliminary safety data of 128 patients who were treated with indoximod or placebo in combination with taxane for metastatic breast cancer (NLG2101 trial)—none developed autoimmune toxicity.^4^ Indoleamine 2,3-dioxygenase is responsible for the degradation of tryptophan to kynurenine in effector T cells and regulatory cells, which leads to downregulation of effector T cells and immune escape.^2^ Indoximod blocks this action, thereby stimulating the immune system. Other immune-boosting agents, such as ICIs, are known to cause autoimmune toxicities, including CNS toxicities.

We believe autoimmunity to be the most likely cause of the patient’s Parkinsonism on the basis of the minimal response achieved with discontinuation of indoximod and Parkinsonism-specific treatment with carbidopa-levodopa and trihexyphenidyl, and near resolution of symptoms after 6 weeks of high-dose corticosteroids. The exact mechanism of indoximod-induced Parkinsonism is unknown at this time. Alternatively, it may be a result of a unique series of metabolic events that involve the compound kynurenine. Of the tryptophan in the brain, 90% is metabolized in the kynurenine pathway. l-kynurenine is consequently metabolized in three alternative metabolites^3,5^: kynurenic acid (neuroprotective), 3-hydroxykynurenine, and quinolinic acid (neurotoxic). Postmortem examinations of patients with PD have demonstrated a reduced l-kynurenine concentration in the frontal cortex, putamen, and substantia nigra compared with healthy control participants. Preclinical data from postmortem and PD mouse models support a shift in tryptophan metabolism toward 3-hydroxykynurenine and, consequently, decreased kynurenic acid compared with healthy control participants, which results in PD.^5^ We speculate that by interfering with tryptophan metabolism, indoximod decreases the concentration of kynurenine and thereby its neuroprotective effect on the brain, which leads to Parkinsonism. Further preclinical, clinical, and biomarker studies are warranted to validate our hypothesis.
